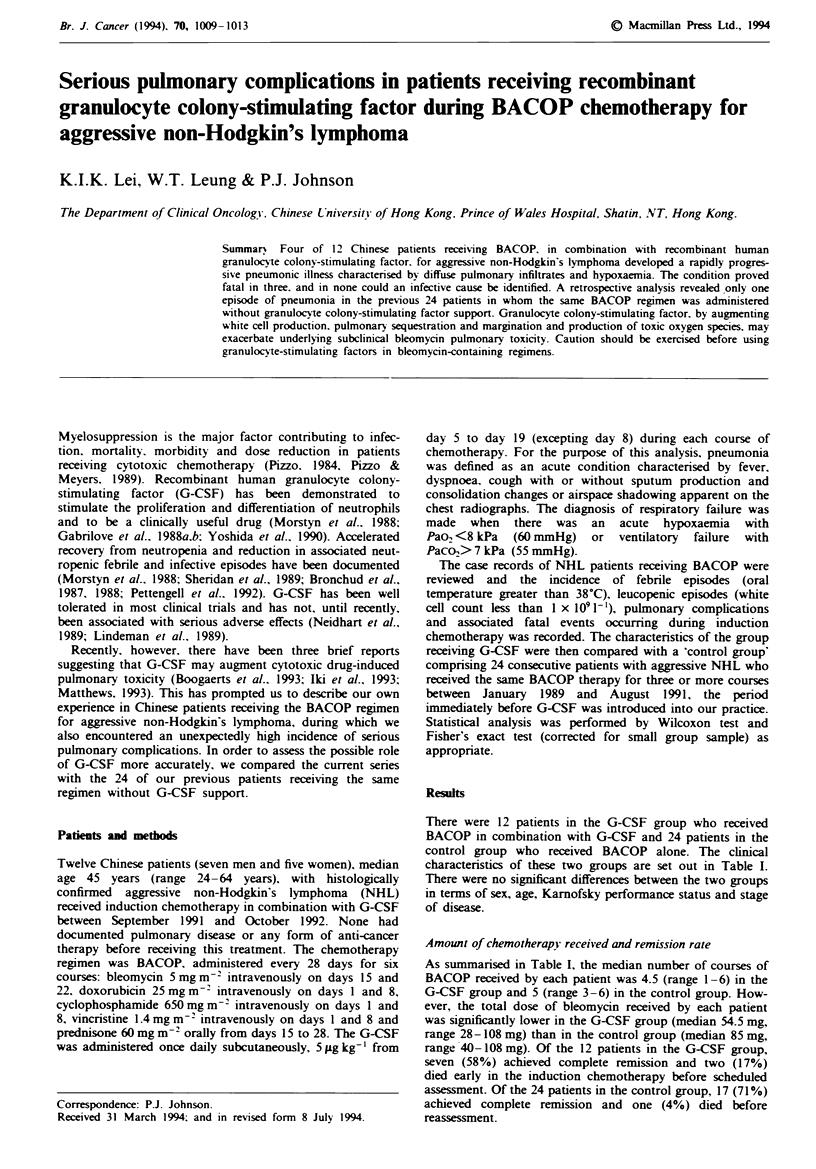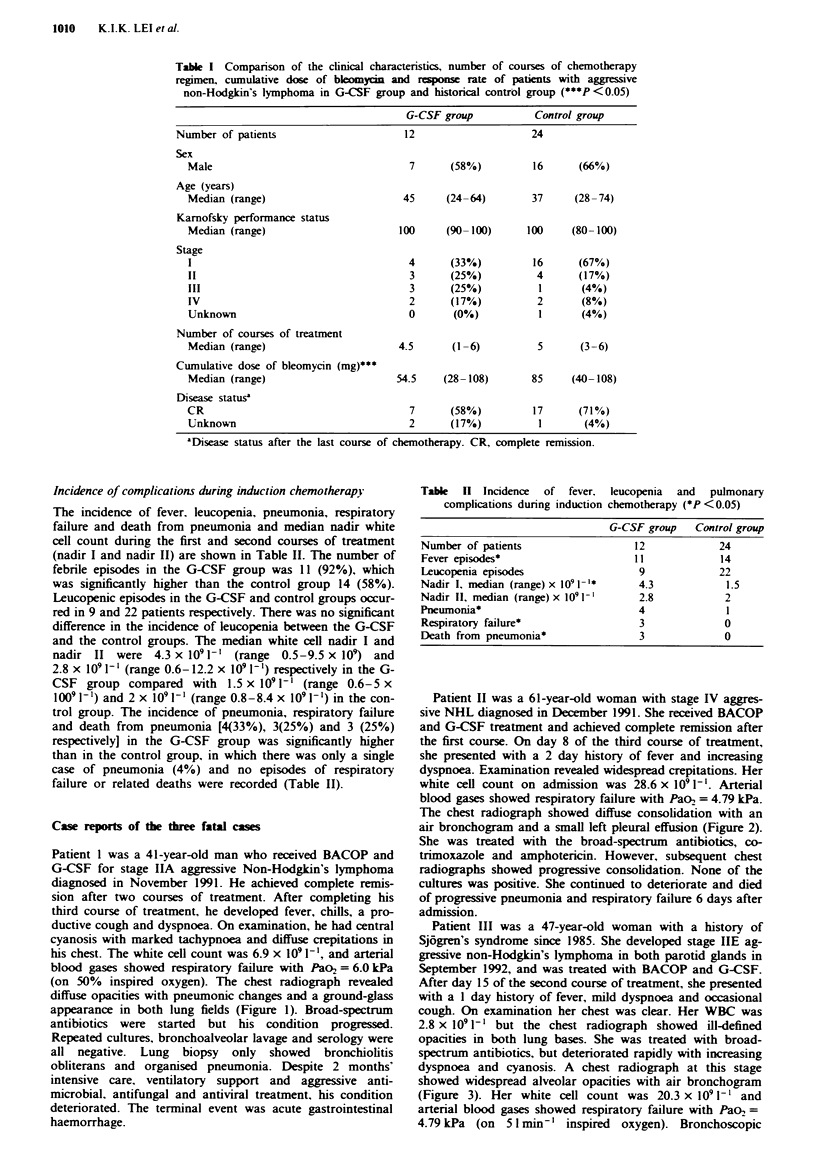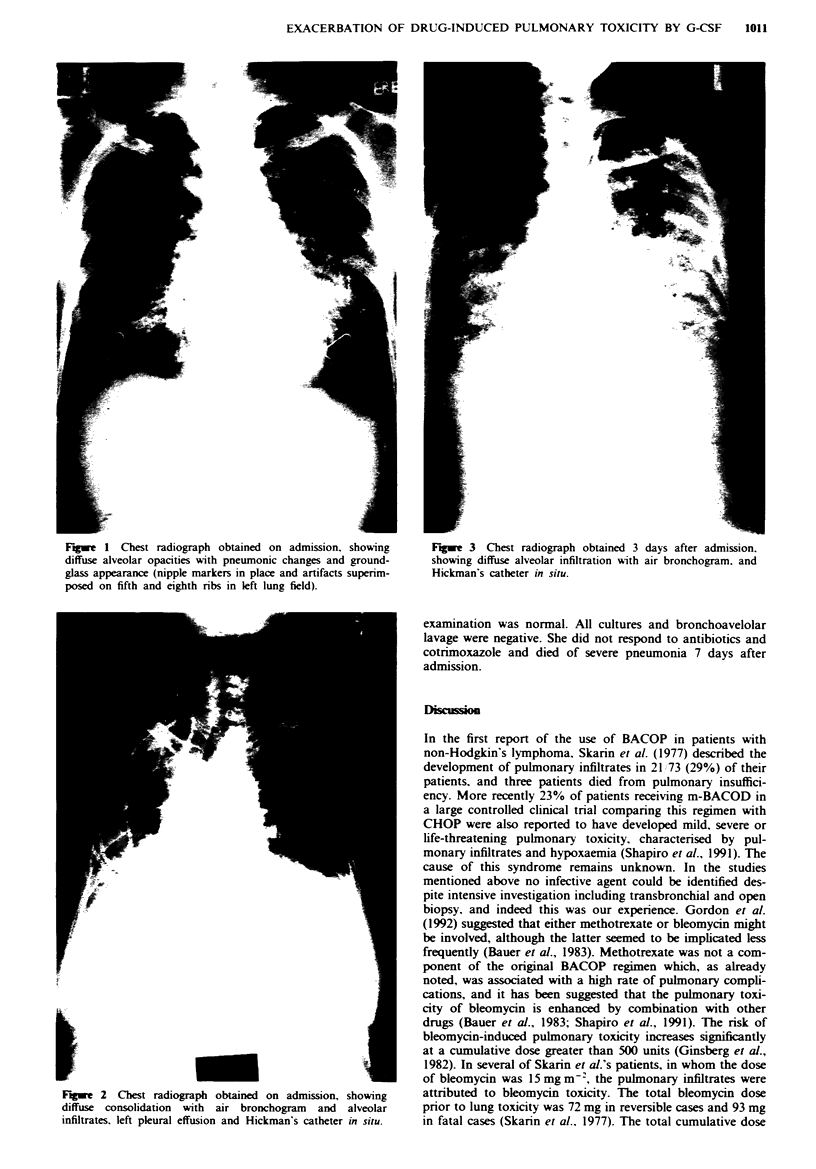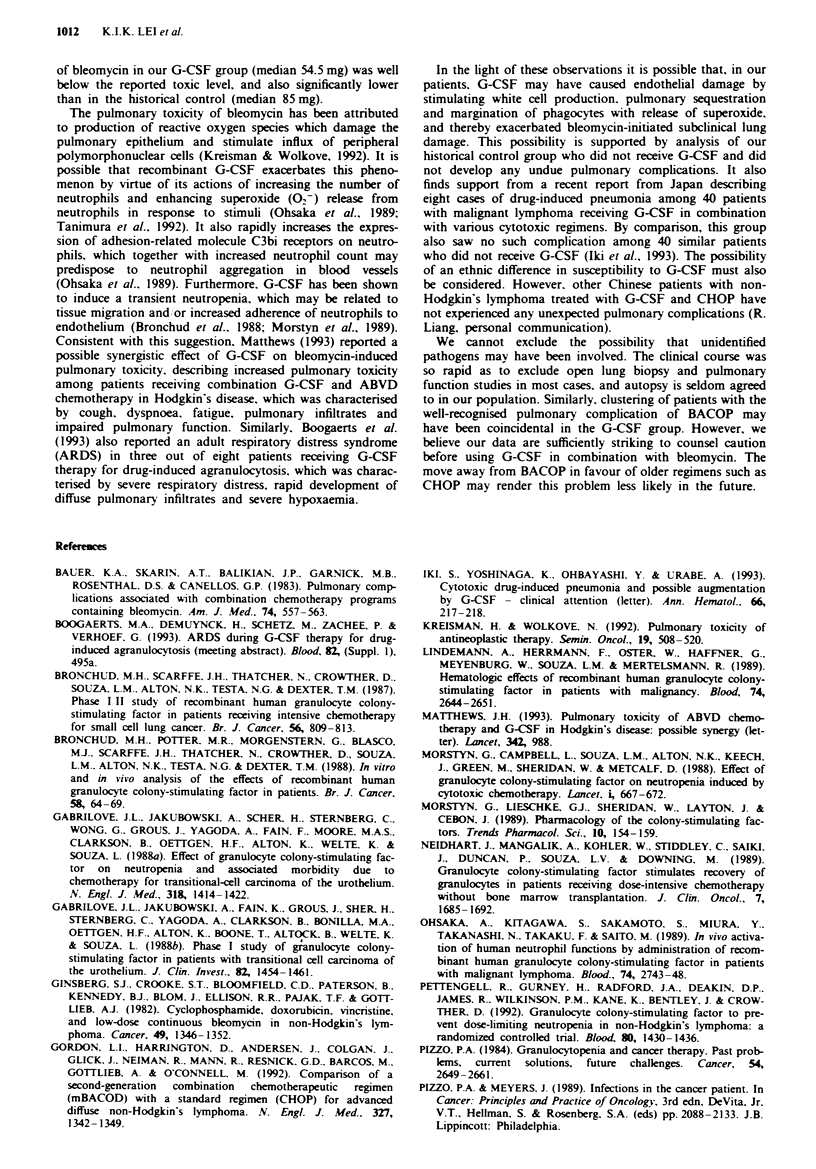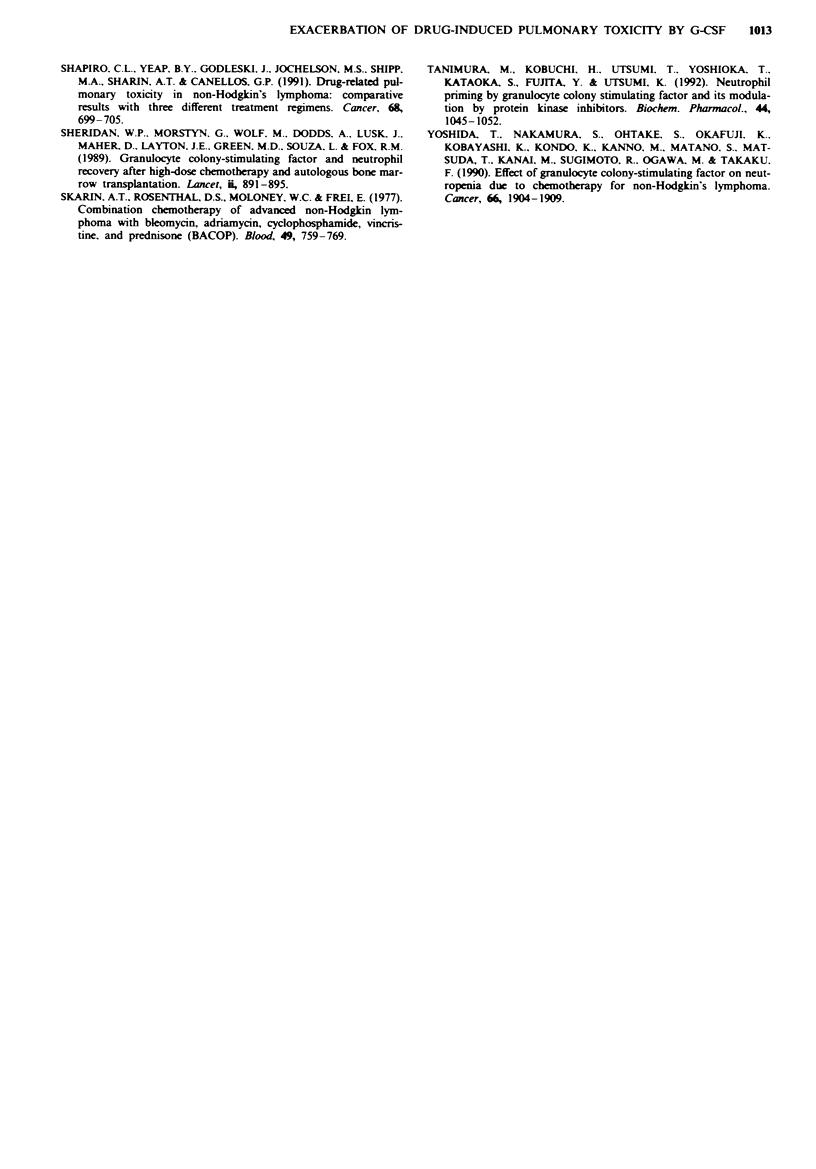# Serious pulmonary complications in patients receiving recombinant granulocyte colony-stimulating factor during BACOP chemotherapy for aggressive non-Hodgkin's lymphoma.

**DOI:** 10.1038/bjc.1994.439

**Published:** 1994-11

**Authors:** K. I. Lei, W. T. Leung, P. J. Johnson

**Affiliations:** Department of Clinical Oncology, Chinese University of Hong Kong, Prince of Wales Hospital, Shatin, NT.

## Abstract

**Images:**


Br. J. Cancer (1994), 70, 1009-1013 © Macmillan Press Ltd., 1994

### Serious pulmonary complications in patients receiving recombinant granulocyte colony-stimulating factor during BACOP chemotherapy for aggressive non-Hodgkin's lymphoma

K.I.K. Lei, W.T. Leung & P.J. Johnson

*The Department of Clinical Oncology, Chinese University of Hong Kong, Prince of Wales Hospital, Shatin, NT, Hong Kong.*

Summary Four of 12 Chinese patients receiving BACOP, in combination with recombinant human granulocyte colony-stimulating factor, for aggressive non-Hodgkin's lymphoma developed a rapidly progressive pneumonic illness characterised by diffuse pulmonary infiltrates and hypoxaemia. The condition proved fatal in three, and in none could an infective cause be identified. A retrospective analysis revealed only one episode of pneumonia in the previous 24 patients in whom the same BACOP regimen was administered without granulocyte colony-stimulating factor support. Granulocyte colony-stimulating factor, by augmenting white cell production, pulmonary sequestration and margination and production of toxic oxygen species, may exacerbate underlying subclinical bleomycin pulmonary toxicity. Caution should be exercised before using granulocyte-stimulating factors in bleomycin-containing regimens.

Myelosuppression is the major factor contributing to infection, mortality, morbidity and dose reduction in patients receiving cytotoxic chemotherapy (Pizzo, 1984, Pizzo & Meyers, 1989). Recombinant human granulocyte colony-stimulating factor (G-CSF) has been demonstrated to stimulate the proliferation and differentiation of neutrophils and to be a clinically useful drug (Morstyn *et al.*, 1988; Gabrilove *et al.*, 1988*a,b*; Yoshida *et al.*, 1990). Accelerated recovery from neutropenia and reduction in associated neutropenic febrile and infective episodes have been documented (Morstyn *et al.*, 1988; Sheridan *et al.*, 1989; Bronchud *et al.*, 1987, 1988; Pettengell *et al.*, 1992). G-CSF has been well tolerated in most clinical trials and has not, until recently, been associated with serious adverse effects (Neidhart *et al.*, 1989; Lindeman *et al.*, 1989).

Recently, however, there have been three brief reports suggesting that G-CSF may augment cytotoxic drug-induced pulmonary toxicity (Boogaerts *et al.*, 1993; Iki *et al.*, 1993; Matthews, 1993). This has prompted us to describe our own experience in Chinese patients receiving the BACOP regimen for aggressive non-Hodgkin's lymphoma, during which we also encountered an unexpectedly high incidence of serious pulmonary complications. In order to assess the possible role of G-CSF more accurately, we compared the current series with the 24 of our previous patients receiving the same regimen without G-CSF support.

### Patients and methods

Twelve Chinese patients (seven men and five women), median age 45 years (range 24-64 years), with histologically confirmed aggressive non-Hodgkin's lymphoma (NHL) received induction chemotherapy in combination with G-CSF between September 1991 and October 1992. None had documented pulmonary disease or any form of anti-cancer therapy before receiving this treatment. The chemotherapy regimen was BACOP, administered every 28 days for six courses: bleomycin 5 mg m$^{-2}$ intravenously on days 15 and 22, doxorubicin 25 mg m$^{-2}$ intravenously on days 1 and 8, cyclophosphamide 650 mg m$^{-2}$ intravenously on days 1 and 8, vincristine 1.4 mg m$^{-2}$ intravenously on days 1 and 8 and prednisone 60 mg m$^{-2}$ orally from days 15 to 28. The G-CSF was administered once daily subcutaneously, 5 $\mu$g kg$^{-1}$ from day 5 to day 19 (excepting day 8) during each course of chemotherapy. For the purpose of this analysis, pneumonia was defined as an acute condition characterised by fever, dyspnoea, cough with or without sputum production and consolidation changes or airspace shadowing apparent on the chest radiographs. The diagnosis of respiratory failure was made when there was an acute hypoxaemia with $Pa\text{O}_2 < 8$ kPa (60 mmHg) or ventilatory failure with $Pa\text{CO}_2 > 7$ kPa (55 mmHg).

The case records of NHL patients receiving BACOP were reviewed and the incidence of febrile episodes (oral temperature greater than 38°C), leucopenic episodes (white cell count less than $1 \times 10^9$ l$^{-1}$), pulmonary complications and associated fatal events occurring during induction chemotherapy was recorded. The characteristics of the group receiving G-CSF were then compared with a 'control group' comprising 24 consecutive patients with aggressive NHL who received the same BACOP therapy for three or more courses between January 1989 and August 1991, the period immediately before G-CSF was introduced into our practice. Statistical analysis was performed by Wilcoxon test and Fisher's exact test (corrected for small group sample) as appropriate.

### Results

There were 12 patients in the G-CSF group who received BACOP in combination with G-CSF and 24 patients in the control group who received BACOP alone. The clinical characteristics of these two groups are set out in Table I. There were no significant differences between the two groups in terms of sex, age, Karnofsky performance status and stage of disease.

### *Amount of chemotherapy received and remission rate*

As summarised in Table I, the median number of courses of BACOP received by each patient was 4.5 (range 1-6) in the G-CSF group and 5 (range 3-6) in the control group. However, the total dose of bleomycin received by each patient was significantly lower in the G-CSF group (median 54.5 mg, range 28-108 mg) than in the control group (median 85 mg, range 40-108 mg). Of the 12 patients in the G-CSF group, seven (58%) achieved complete remission and two (17%) died early in the induction chemotherapy before scheduled assessment. Of the 24 patients in the control group, 17 (71%) achieved complete remission and one (4%) died before reassessment.

Correspondence: P.J. Johnson.
Received 31 March 1994; and in revised form 8 July 1994.

**Table I** Comparison of the clinical characteristics, number of courses of chemotherapy regimen, cumulative dose of bleomycin and response rate of patients with aggressive non-Hodgkin's lymphoma in G-CSF group and historical control group (***$P < 0.05$)

| | *G-CSF group* | | *Control group* | |
|---|---|---|---|---|
| Number of patients | 12 | | 24 | |
| Sex | | | | |
| Male | 7 | (58%) | 16 | (66%) |
| Age (years) | | | | |
| Median (range) | 45 | (24-64) | 37 | (28-74) |
| Karnofsky performance status | | | | |
| Median (range) | 100 | (90-100) | 100 | (80-100) |
| Stage | | | | |
| I | 4 | (33%) | 16 | (67%) |
| II | 3 | (25%) | 4 | (17%) |
| III | 3 | (25%) | 1 | (4%) |
| IV | 2 | (17%) | 2 | (8%) |
| Unknown | 0 | (0%) | 1 | (4%) |
| Number of courses of treatment | | | | |
| Median (range) | 4.5 | (1-6) | 5 | (3-6) |
| Cumulative dose of bleomycin (mg)*** | | | | |
| Median (range) | 54.5 | (28-108) | 85 | (40-108) |
| Disease status[a] | | | | |
| CR | 7 | (58%) | 17 | (71%) |
| Unknown | 2 | (17%) | 1 | (4%) |

[a]Disease status after the last course of chemotherapy. CR, complete remission.

*Incidence of complications during induction chemotherapy*

The incidence of fever, leucopenia, pneumonia, respiratory failure and death from pneumonia and median nadir white cell count during the first and second courses of treatment (nadir I and nadir II) are shown in Table II. The number of febrile episodes in the G-CSF group was 11 (92%), which was significantly higher than the control group 14 (58%). Leucopenic episodes in the G-CSF and control groups occurred in 9 and 22 patients respectively. There was no significant difference in the incidence of leucopenia between the G-CSF and the control groups. The median white cell nadir I and nadir II were $4.3 \times 10^9 l^{-1}$ (range $0.5-9.5 \times 10^9$) and $2.8 \times 10^9 l^{-1}$ (range $0.6-12.2 \times 10^9 l^{-1}$) respectively in the G-CSF group compared with $1.5 \times 10^9 l^{-1}$ (range $0.6-5 \times 100^9 l^{-1}$) and $2 \times 10^9 l^{-1}$ (range $0.8-8.4 \times 10^9 l^{-1}$) in the control group. The incidence of pneumonia, respiratory failure and death from pneumonia [4(33%), 3(25%) and 3 (25%) respectively] in the G-CSF group was significantly higher than in the control group, in which there was only a single case of pneumonia (4%) and no episodes of respiratory failure or related deaths were recorded (Table II).

**Table II** Incidence of fever, leucopenia and pulmonary complications during induction chemotherapy (*$P < 0.05$)

| | *G-CSF group* | *Control group* |
|---|---|---|
| Number of patients | 12 | 24 |
| Fever episodes* | 11 | 14 |
| Leucopenia episodes | 9 | 22 |
| Nadir I, median (range) $\times 10^9 l^{-1}$* | 4.3 | 1.5 |
| Nadir II, median (range) $\times 10^9 l^{-1}$ | 2.8 | 2 |
| Pneumonia* | 4 | 1 |
| Respiratory failure* | 3 | 0 |
| Death from pneumonia* | 3 | 0 |

### Case reports of the three fatal cases

Patient 1 was a 41-year-old man who received BACOP and G-CSF for stage IIA aggressive Non-Hodgkin's lymphoma diagnosed in November 1991. He achieved complete remission after two courses of treatment. After completing his third course of treatment, he developed fever, chills, a productive cough and dyspnoea. On examination, he had central cyanosis with marked tachypnoea and diffuse crepitations in his chest. The white cell count was $6.9 \times 10^9 l^{-1}$, and arterial blood gases showed respiratory failure with $Pa_{O_2} = 6.0$ kPa (on 50% inspired oxygen). The chest radiograph revealed diffuse opacities with pneumonic changes and a ground-glass appearance in both lung fields (Figure 1). Broad-spectrum antibiotics were started but his condition progressed. Repeated cultures, bronchoalveolar lavage and serology were all negative. Lung biopsy only showed bronchiolitis obliterans and organised pneumonia. Despite 2 months' intensive care, ventilatory support and aggressive antimicrobial, antifungal and antiviral treatment, his condition deteriorated. The terminal event was acute gastrointestinal haemorrhage.

Patient II was a 61-year-old woman with stage IV aggressive NHL diagnosed in December 1991. She received BACOP and G-CSF treatment and achieved complete remission after the first course. On day 8 of the third course of treatment, she presented with a 2 day history of fever and increasing dyspnoea. Examination revealed widespread crepitations. Her white cell count on admission was $28.6 \times 10^9 l^{-1}$. Arterial blood gases showed respiratory failure with $Pa_{O_2} = 4.79$ kPa. The chest radiograph showed diffuse consolidation with an air bronchogram and a small left pleural effusion (Figure 2). She was treated with the broad-spectrum antibiotics, co-trimoxazole and amphotericin. However, subsequent chest radiographs showed progressive consolidation. None of the cultures was positive. She continued to deteriorate and died of progressive pneumonia and respiratory failure 6 days after admission.

Patient III was a 47-year-old woman with a history of Sjögren's syndrome since 1985. She developed stage IIE aggressive non-Hodgkin's lymphoma in both parotid glands in September 1992, and was treated with BACOP and G-CSF. After day 15 of the second course of treatment, she presented with a 1 day history of fever, mild dyspnoea and occasional cough. On examination her chest was clear. Her WBC was $2.8 \times 10^9 l^{-1}$ but the chest radiograph showed ill-defined opacities in both lung bases. She was treated with broad-spectrum antibiotics, but deteriorated rapidly with increasing dyspnoea and cyanosis. A chest radiograph at this stage showed widespread alveolar opacities with air bronchogram (Figure 3). Her white cell count was $20.3 \times 10^9 l^{-1}$ and arterial blood gases showed respiratory failure with $Pa_{O_2} =$ 4.79 kPa (on $5 l min^{-1}$ inspired oxygen). Bronchoscopic

**Figure 1** Chest radiograph obtained on admission, showing diffuse alveolar opacities with pneumonic changes and ground-glass appearance (nipple markers in place and artifacts superimposed on fifth and eighth ribs in left lung field).

**Figure 3** Chest radiograph obtained 3 days after admission, showing diffuse alveolar infiltration with air bronchogram, and Hickman's catheter *in situ*.

**Figure 2** Chest radiograph obtained on admission, showing diffuse consolidation with air bronchogram and alveolar infiltrates, left pleural effusion and Hickman's catheter *in situ*.

examination was normal. All cultures and bronchoavelolar lavage were negative. She did not respond to antibiotics and cotrimoxazole and died of severe pneumonia 7 days after admission.

### Discussion

In the first report of the use of BACOP in patients with non-Hodgkin's lymphoma, Skarin *et al.* (1977) described the development of pulmonary infiltrates in 21/73 (29%) of their patients, and three patients died from pulmonary insufficiency. More recently 23% of patients receiving m-BACOD in a large controlled clinical trial comparing this regimen with CHOP were also reported to have developed mild, severe or life-threatening pulmonary toxicity, characterised by pulmonary infiltrates and hypoxaemia (Shapiro *et al.*, 1991). The cause of this syndrome remains unknown. In the studies mentioned above no infective agent could be identified despite intensive investigation including transbronchial and open biopsy, and indeed this was our experience. Gordon *et al.* (1992) suggested that either methotrexate or bleomycin might be involved, although the latter seemed to be implicated less frequently (Bauer *et al.*, 1983). Methotrexate was not a component of the original BACOP regimen which, as already noted, was associated with a high rate of pulmonary complications, and it has been suggested that the pulmonary toxicity of bleomycin is enhanced by combination with other drugs (Bauer *et al.*, 1983; Shapiro *et al.*, 1991). The risk of bleomycin-induced pulmonary toxicity increases significantly at a cumulative dose greater than 500 units (Ginsberg *et al.*, 1982). In several of Skarin *et al.*'s patients, in whom the dose of bleomycin was 15 mg $m^{-2}$, the pulmonary infiltrates were attributed to bleomycin toxicity. The total bleomycin dose prior to lung toxicity was 72 mg in reversible cases and 93 mg in fatal cases (Skarin *et al.*, 1977). The total cumulative dose of bleomycin in our G-CSF group (median 54.5 mg) was well below the reported toxic level, and also significantly lower than in the historical control (median 85 mg).

The pulmonary toxicity of bleomycin has been attributed to production of reactive oxygen species which damage the pulmonary epithelium and stimulate influx of peripheral polymorphonuclear cells (Kreisman & Wolkove, 1992). It is possible that recombinant G-CSF exacerbates this phenomenon by virtue of its actions of increasing the number of neutrophils and enhancing superoxide ($O_2^-$) release from neutrophils in response to stimuli (Ohsaka *et al.*, 1989; Tanimura *et al.*, 1992). It also rapidly increases the expression of adhesion-related molecule C3bi receptors on neutrophils, which together with increased neutrophil count may predispose to neutrophil aggregation in blood vessels (Ohsaka *et al.*, 1989). Furthermore, G-CSF has been shown to induce a transient neutropenia, which may be related to tissue migration and/or increased adherence of neutrophils to endothelium (Bronchud *et al.*, 1988; Morstyn *et al.*, 1989). Consistent with this suggestion, Matthews (1993) reported a possible synergistic effect of G-CSF on bleomycin-induced pulmonary toxicity, describing increased pulmonary toxicity among patients receiving combination G-CSF and ABVD chemotherapy in Hodgkin's disease, which was characterised by cough, dyspnoea, fatigue, pulmonary infiltrates and impaired pulmonary function. Similarly, Boogaerts *et al.* (1993) also reported an adult respiratory distress syndrome (ARDS) in three out of eight patients receiving G-CSF therapy for drug-induced agranulocytosis, which was characterised by severe respiratory distress, rapid development of diffuse pulmonary infiltrates and severe hypoxaemia.

In the light of these observations it is possible that, in our patients, G-CSF may have caused endothelial damage by stimulating white cell production, pulmonary sequestration and margination of phagocytes with release of superoxide, and thereby exacerbated bleomycin-initiated subclinical lung damage. This possibility is supported by analysis of our historical control group who did not receive G-CSF and did not develop any undue pulmonary complications. It also finds support from a recent report from Japan describing eight cases of drug-induced pneumonia among 40 patients with malignant lymphoma receiving G-CSF in combination with various cytotoxic regimens. By comparison, this group also saw no such complication among 40 similar patients who did not receive G-CSF (Iki *et al.*, 1993). The possibility of an ethnic difference in susceptibility to G-CSF must also be considered. However, other Chinese patients with non-Hodgkin's lymphoma treated with G-CSF and CHOP have not experienced any unexpected pulmonary complications (R. Liang, personal communication).

We cannot exclude the possibility that unidentified pathogens may have been involved. The clinical course was so rapid as to exclude open lung biopsy and pulmonary function studies in most cases, and autopsy is seldom agreed to in our population. Similarly, clustering of patients with the well-recognised pulmonary complication of BACOP may have been coincidental in the G-CSF group. However, we believe our data are sufficiently striking to counsel caution before using G-CSF in combination with bleomycin. The move away from BACOP in favour of older regimens such as CHOP may render this problem less likely in the future.

### References

BAUER, K.A., SKARIN, A.T., BALIKIAN, J.P., GARNICK, M.B., ROSENTHAL, D.S. & CANELLOS, G.P. (1983). Pulmonary complications associated with combination chemotherapy programs containing bleomycin. *Am. J. Med.*, **74**, 557-563.

BOOGAERTS, M.A., DEMUYNCK, H., SCHETZ, M., ZACHEE, P. & VERHOEF, G. (1993). ARDS during G-CSF therapy for drug-induced agranulocytosis (meeting abstract). *Blood*, **82**, (Suppl. 1), 495a.

BRONCHUD, M.H., SCARFFE, J.H., THATCHER, N., CROWTHER, D., SOUZA, L.M., ALTON, N.K., TESTA, N.G. & DEXTER, T.M. (1987). Phase I/II study of recombinant human granulocyte colony-stimulating factor in patients receiving intensive chemotherapy for small cell lung cancer. *Br. J. Cancer*, **56**, 809-813.

BRONCHUD, M.H., POTTER, M.R., MORGENSTERN, G., BLASCO, M.J., SCARFFE, J.H., THATCHER, N., CROWTHER, D., SOUZA, L.M., ALTON, N.K., TESTA, N.G. & DEXTER, T.M. (1988). *In vitro* and *in vivo* analysis of the effects of recombinant human granulocyte colony-stimulating factor in patients. *Br. J. Cancer*, **58**, 64-69.

GABRILOVE, J.L., JAKUBOWSKI, A., SCHER, H., STERNBERG, C., WONG, G., GROUS, J., YAGODA, A., FAIN, F., MOORE, M.A.S., CLARKSON, B., OETTGEN, H.F., ALTON, K., WELTE, K. & SOUZA, L. (1988*a*). Effect of granulocyte colony-stimulating factor on neutropenia and associated morbidity due to chemotherapy for transitional-cell carcinoma of the urothelium. *N. Engl. J. Med.*, **318**, 1414-1422.

GABRILOVE, J.L., JAKUBOWSKI, A., FAIN, K., GROUS, J., SHER, H., STERNBERG, C., YAGODA, A., CLARKSON, B., BONILLA, M.A., OETTGEN, H.F., ALTON, K., BOONE, T., ALTOCK, B., WELTE, K. & SOUZA, L. (1988*b*). Phase I study of granulocyte colony-stimulating factor in patients with transitional cell carcinoma of the urothelium. *J. Clin. Invest.*, **82**, 1454-1461.

GINSBERG, S.J., CROOKE, S.T., BLOOMFIELD, C.D., PATERSON, B., KENNEDY, B.J., BLOM, J., ELLISON, R.R., PAJAK, T.F. & GOTTLIEB, A.J. (1982). Cyclophosphamide, doxorubicin, vincristine, and low-dose continuous bleomycin in non-Hodgkin's lymphoma. *Cancer*, **49**, 1346-1352.

GORDON, L.I., HARRINGTON, D., ANDERSEN, J., COLGAN, J., GLICK, J., NEIMAN, R., MANN, R., RESNICK, G.D., BARCOS, M., GOTTLIEB, A. & O'CONNELL, M. (1992). Comparison of a second-generation combination chemotherapeutic regimen (mBACOD) with a standard regimen (CHOP) for advanced diffuse non-Hodgkin's lymphoma. *N. Engl. J. Med.*, **327**, 1342-1349.

IKI, S., YOSHINAGA, K., OHBAYASHI, Y. & URABE, A. (1993). Cytotoxic drug-induced pneumonia and possible augmentation by G-CSF - clinical attention (letter). *Ann. Hematol.*, **66**, 217-218.

KREISMAN, H. & WOLKOVE, N. (1992). Pulmonary toxicity of antineoplastic therapy. *Semin. Oncol.*, **19**, 508-520.

LINDEMANN, A., HERRMANN, F., OSTER, W., HAFFNER, G., MEYENBURG, W., SOUZA, L.M. & MERTELSMANN, R. (1989). Hematologic effects of recombinant human granulocyte colony-stimulating factor in patients with malignancy. *Blood*, **74**, 2644-2651.

MATTHEWS, J.H. (1993). Pulmonary toxicity of ABVD chemotherapy and G-CSF in Hodgkin's disease: possible synergy (letter). *Lancet*, **342**, 988.

MORSTYN, G., CAMPBELL, L., SOUZA, L.M., ALTON, N.K., KEECH, J., GREEN, M., SHERIDAN, W. & METCALF, D. (1988). Effect of granulocyte colony-stimulating factor on neutropenia induced by cytotoxic chemotherapy. *Lancet*, **i**, 667-672.

MORSTYN, G., LIESCHKE, G.J., SHERIDAN, W., LAYTON, J. & CEBON, J. (1989). Pharmacology of the colony-stimulating factors. *Trends Pharmacol. Sci.*, **10**, 154-159.

NEIDHART, J., MANGALIK, A., KOHLER, W., STIDDLEY, C., SAIKI, J., DUNCAN, P., SOUZA, L.V. & DOWNING, M. (1989). Granulocyte colony-stimulating factor stimulates recovery of granulocytes in patients receiving dose-intensive chemotherapy without bone marrow transplantation. *J. Clin. Oncol.*, **7**, 1685-1692.

OHSAKA, A., KITAGAWA, S., SAKAMOTO, S., MIURA, Y., TAKANASHI, N., TAKAKU, F. & SAITO, M. (1989). *In vivo* activation of human neutrophil functions by administration of recombinant human granulocyte colony-stimulating factor in patients with malignant lymphoma. *Blood.*, **74**, 2743-48.

PETTENGELL, R., GURNEY, H., RADFORD, J.A., DEAKIN, D.P., JAMES, R., WILKINSON, P.M., KANE, K., BENTLEY, J. & CROWTHER, D. (1992). Granulocyte colony-stimulating factor to prevent dose-limiting neutropenia in non-Hodgkin's lymphoma: a randomized controlled trial. *Blood*, **80**, 1430-1436.

PIZZO, P.A. (1984). Granulocytopenia and cancer therapy. Past problems, current solutions, future challenges. *Cancer*, **54**, 2649-2661.

PIZZO, P.A. & MEYERS, J. (1989). Infections in the cancer patient. In *Cancer: Principles and Practice of Oncology*, 3rd edn. DeVita, Jr. V.T., Hellman, S. & Rosenberg, S.A. (eds) pp. 2088-2133. J.B. Lippincott: Philadelphia.

SHAPIRO, C.L., YEAP, B.Y., GODLESKI, J., JOCHELSON, M.S., SHIPP, M.A., SHARIN, A.T. & CANELLOS, G.P. (1991). Drug-related pulmonary toxicity in non-Hodgkin's lymphoma: comparative results with three different treatment regimens. *Cancer*, **68**, 699–705.

SHERIDAN, W.P., MORSTYN, G., WOLF, M., DODDS, A., LUSK, J., MAHER, D., LAYTON, J.E., GREEN, M.D., SOUZA, L. & FOX, R.M. (1989). Granulocyte colony-stimulating factor and neutrophil recovery after high-dose chemotherapy and autologous bone marrow transplantation. *Lancet*, **ii**, 891–895.

SKARIN, A.T., ROSENTHAL, D.S., MOLONEY, W.C. & FREI, E. (1977). Combination chemotherapy of advanced non-Hodgkin lymphoma with bleomycin, adriamycin, cyclophosphamide, vincristine, and prednisone (BACOP). *Blood*, **49**, 759–769.

TANIMURA, M., KOBUCHI, H., UTSUMI, T., YOSHIOKA, T., KATAOKA, S., FUJITA, Y. & UTSUMI, K. (1992). Neutrophil priming by granulocyte colony stimulating factor and its modulation by protein kinase inhibitors. *Biochem. Pharmacol.*, **44**, 1045–1052.

YOSHIDA, T., NAKAMURA, S., OHTAKE, S., OKAFUJI, K., KOBAYASHI, K., KONDO, K., KANNO, M., MATANO, S., MATSUDA, T., KANAI, M., SUGIMOTO, R., OGAWA, M. & TAKAKU, F. (1990). Effect of granulocyte colony-stimulating factor on neutropenia due to chemotherapy for non-Hodgkin's lymphoma. *Cancer*, **66**, 1904–1909.